# Comparative analysis of bio-based plasticizers: biocompatibility, plasticizing mechanisms, and molecular dynamics insights†

**DOI:** 10.1039/d4ra07258h

**Published:** 2025-02-10

**Authors:** Li Gao, Rui Yuan, Lijuan Qiao, Chang Tu, Rui Tan, Shiai Xu

**Affiliations:** a Shanghai Key Laboratory of Advanced Polymeric Materials, Key Laboratory for Ultrafine Materials of Ministry of Education, School of Materials Science and Engineering, East China University of Science and Technology Shanghai 200237 China; b Salt Lake Chemical Engineering Research Complex, Qinghai University Xining 810016 China; c Research Center of Basic Medical Science, Medical College, Qinghai University Xining 810016 China

## Abstract

The advantages of bio-based plasticizers and the differences in their biotoxicity and plasticizing mechanisms compared to phthalate plasticizers have rarely been systematically investigated. Epoxidized soybean oil (ESO), triphenyl phosphate (TCP), and acetyl tributyl citrate (ATBC) were specifically chosen for a rigorous comparative analysis with diocty phthalate (DOP), employing a blend of toxicological studies, characterization methodologies and molecular dynamics (MD) simulations. Based on the blood routine indicators and liver tissue pathology analysis in SD rats, the biocompatibility ranking is as follows: ESO > ATBC > TCP > DOP. When the plasticizer content is 40 wt%, ATBC/PVC and ESO/PVC exhibit superior elongation at break compared to DOP/PVC. MD results indicate that ATBC, ESO, TCP, and DOP can all spontaneously disperse in PVC. Among them, ESO exhibits the highest compatibility with PVC attributed to the interaction forces. For ESO/PVC, interactions include electrostatic forces between polar groups, van der Waals forces, and the entangling of alkyl chains. For ATBC/PVC, the interaction is primarily due to the hydrophobic alkyl chains entangling with PVC through hydrophobic interactions. These observations have been corroborated by MD results, providing additional insights into the underlying microscopic mechanisms. This study offers theoretical support for the broader utilization of environmentally friendly plasticizers.

## Abstract 2

Advantages of bio-based plasticizers and plasticizer mechanics compared to phthalate plasticizers.

## Graphical abstract

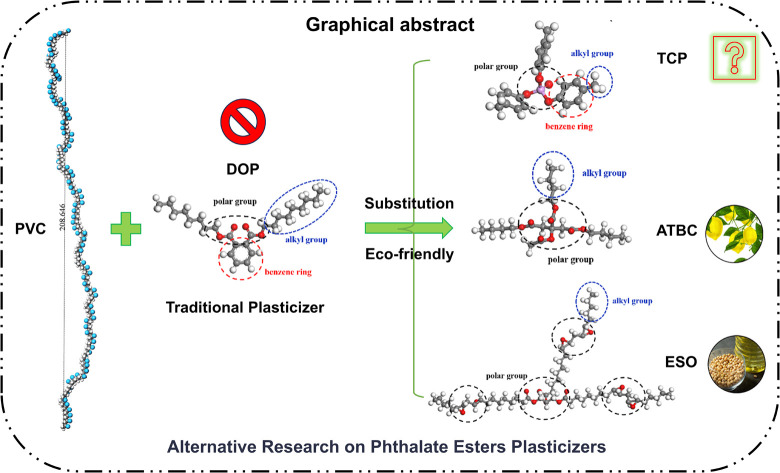

## 1 Introduction

Polyvinyl chloride (PVC) stands out as a paramount thermoplastic, enjoying widespread utilization across various sectors, including piping, medical facilities, and packaging materials.^1–4^ Because of the presence of C–Cl bonds within PVC chains, neat PVC is rigid and difficult to process.^5^ Therefore, it is necessary to introduce large quantities of plasticizers into PVC resin to achieve the desired flexibility.

Phthalate plasticizers (PAEs), such as dioctyl phthalate (DOP), are widely employed as commercial plasticizers for PVC, accounting for over 80% of total plasticizer usage.^6^ However, studies have revealed that PAEs are prone to migrating to the surface of the PVC matrix over extended periods of service, particularly in environments involving water and oil.^7–9^ PAEs have hepatotoxicity and carcinogenicity in mice. This migration of PAEs not only damages the properties of the PVC material, but also poses a threat to human health. Hence their application is restricted in specific domains, including medical facilities and children's supplies. Due to health concerns, extensive research has been conducted on alternatives to plasticized PVC formulations.^10,11^

Many researchers are committed to developing environment-friendly biobased plasticizers, such as citric acid, fatty acid esters, plant oils, *etc.*, as substitutes for PAEs.^12–17^ Arya *et al.* used triphenyl phosphate (TCP) as a plasticizer and flame retardant to minimize residual solvents and improve the performance of poly(styrene)-*p*-xylene coatings.^18^ Liu *et al.* prepared ethyl cellulose (EC) films using epoxidized soybean oil (ESO), which exhibited better thermal stability, mechanical properties, non-flammability, and lower water vapor permeability.^19^ Guo *et al.* used four novel ESO plasticizers to improve the toughness of PVC and achieved a significant improvement in its mechanical properties.^20^ Sawada *et al.* fabricated flat films of poly(vinylidene fluoride) with acetyl tributyl citrate (ATBC).^21^ Although current research results show that environment friendly plasticizers such as ATBC, ESO and TCP can improve the plasticity of resins, few researchers have conducted controlled studies on these plasticizers, especially on the microscopic plasticizing mechanism, plasticizing efficiency, solvent resistance and so on. And these fundamental investigations play a crucial role in selecting and modifying environmentally friendly plasticizers.

Molecular dynamics (MD) simulations prove invaluable in elucidating the structures and dynamic processes of various components within PVC composites, crucial determinants of the material's mechanical properties and stability.^22–26^ Many of these aspects are challenging to monitor experimentally. Lin *et al.* delved into the correlation between the structure of PVC and its thermal stability through a combination of experimental analysis and MD simulations.^27^ Yang *et al.* investigated the diffusion law of diesel compositions across PVC and polyvinyl alcohol films.^28^ In a similar vein, Gao *et al.* explored the impact of various plasticizers on the rheological performances of asphalt and elucidated the underlying mechanisms by MD simulations.^29^ In addition, in a previous study, we investigated the microscopic plasticizing mechanism of citrate plasticizers in polyvinyl chloride resins using MD simulations and revealed the mechanism by which acetylation modifications in the citrate molecule affect the plasticizing efficiency.^30^ Hence, MD simulations serve as a valuable tool for unveiling internal microscopic changes that may be challenging to observe experimentally.

In this study, the biotoxicity of four plasticizers was assessed *via* gavage in SD rats to evaluate environmental impact of ATBC, ESO, TCP, and DOP. The mechanical and stability properties of plasticizer/PVC composites were evaluated using a universal testing machine and migration resistance test to compare their overall performance. Additionally, MD was employed to investigate the microscopic plasticizing mechanism, PVC resin compatibility, and tensile failure mechanism of the four plasticizers mentioned above.

## 2 Experimental

### 2.1 Biological toxicity of ATBC, ESO, TCP, DOP

In order to evaluate the toxicity of four plasticizers-ATBC, ESO, TCP, and DOP-19 SPF clean-grade SD rats (10 females and 9 males) aged 4–6 weeks were selected. The experiment was approved by the Medical Science Research Ethics Committee of the Medical College of Qinghai University. All SD rats were purchased from the Lanzhou Veterinary Research Institute of the Chinese Academy of Agricultural Sciences. The approval number for this study is SCXK (Gan) 2020-0002. The initial body weight of the subjects was recorded. The rats were divided into five groups: control, ATBC, ESO, TCP, and DOP. The control group consisted of 2 females and 1 male, while the other groups each had 2 females and 2 males. During the normal feeding period (timed and quantified), SD rats in the control group were administered carboxymethyl cellulose sodium (0.5%) *via* gavage, while SD rats in the other groups received plasticizer/carboxymethyl cellulose sodium (2500 mg kg^−1^). Each rat was gavaged with 1.5 ml of the solution daily at a fixed time for 28 days. Monitor the daily survival status and weight of each rat on days 7, 14, and 28.

**Fig. 1:**
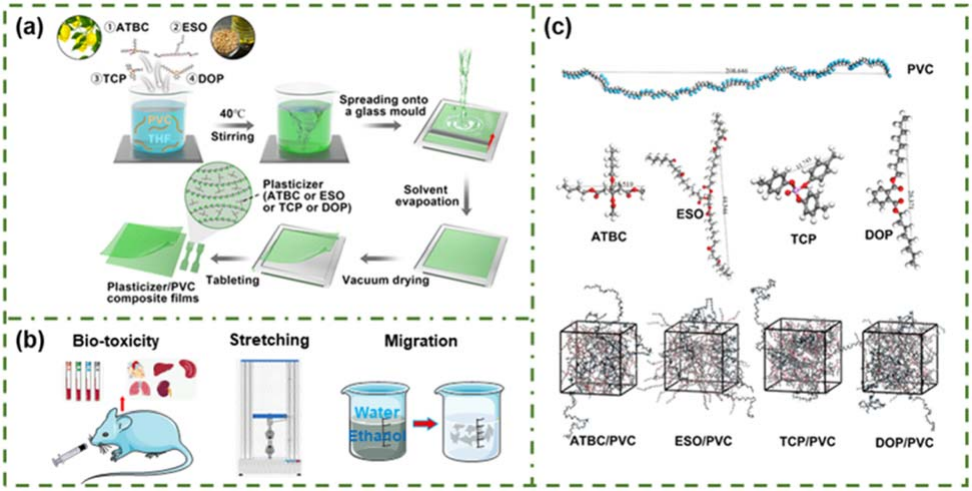
(a) The synthetic procedure of PVC, ATBC/PVC, ESO/PVC, TCP/PVC and DOP/PVC films. (b) Biological toxicity of four plasticizers and performance testing of PVC based composites. (c) Molecular model (PVC, ATBC, ESO, TCP and DOP) and MD system of S_ATBC/PVC_, S_ESO/PVC_, S_TCP/PVC_ and S_DOP/PVC_.

After 28 days of gastric gavage in rats, fasting overnight, anesthesia was induced by intraperitoneal injection of 0.7 ml/100 g pentobarbital sodium based on body weight. Blood samples were collected *via* the abdominal aorta for biochemical and immunological analyses. Following blood collection, the rats were euthanized, and organs such as heart, liver, spleen, lungs, kidneys, brain, testes, and ovary were rapidly fixed in formalin solution for histopathological examination using HE staining (Fig. 1(b)).^31^ Data analysis was conducted using one-way analysis of variance (ANOVA) with SPSS27 software to test for differences.

### 2.2 Preparation of plasticizer/PVC composites

PVC-based composites were fabricated *via* solvent casting (Fig. 1(a)). Initially, a specific amount of PVC and plasticizers (ATBC, ESO, TCP, and DOP) were added to THF and magnetically stirred until uniformly mixed. The resulting mixture was then poured onto a glass mold and left to set overnight, followed by drying at 50 °C for 8 h. As illustrated in Fig. S1,† the flatness and roughness of both PVC and plasticizer/PVC films were evaluated. It was observed that compared to pure PVC, the surface of the plasticized PVC films exhibited significantly smoother characteristics. For subsequent performance evaluations (Fig. 1(b)), the obtained films underwent tableting treatment.

### 2.3 Plasticizer migration

This experiment was conducted according to ASTM D1239-98, utilizing a 50 × 50 mm square specimen. The migration medium consisted of 100% deionized water and 50% (v/v) ethanol in deionized water. The degree of migration was determined using the following formula:^32^
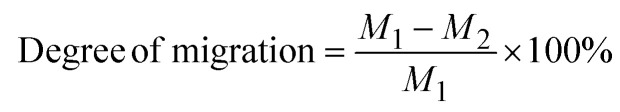
Here, *M*_1_ and *M*_2_ represent the mass of PVC film before and after migration test, respectively.

### 2.4 Mechanical test

The mechanical test was conducted according to ASTM D-638 standards. The universal testing machine (WDW-200, China) was operated at a tensile rate of 100 mm min^−1^ and the tests were performed at room temperature. Detailed descriptions of other experimental methods were shown in the ESI.†

### 2.5 MD simulations

MD simulations were conducted to study the micro plasticization mechanisms of ATBC, ESO, TCP, and DOP, along with their corresponding tensile failure mechanisms of PVC-based composites. The COMPASS force field was used to construct four simulation systems (S_TEC/PVC_, S_ATEC/PVC_, S_TBC/PVC_, and S_ATBC/PVC_) through all-atom MD simulations.^33^ As depicted in Fig. 1(c), the PVC chain comprised 80 vinyl chloride monomers. For the ATBC/PVC, ESO/PVC, TCP/PVC, and DOP/PVC systems, four PVC chains were included. To maintain a 1 : 1 mass ratio of plasticizers to PVC in the simulation setup, the number of introduced plasticizers was 61 (ATBC), 32 (ESO), 67 (TCP), and 63 (DOP), as shown in Table S1.†

The initial configuration of PVC-based systems underwent optimization using a smart algorithm and three-dimensional boundary conditions. Long-range electrostatics were computed using the Ewald method, while van der Waals interactions were analyzed using the atom-based method.^34^ Subsequently, each system underwent thermal annealing through 50 NVT simulation cycles within a temperature range of 300 K to 500 K. Following this, the systems underwent NVT dynamic equilibration for 10 ns, employing a time step of 1 fs at 298 K (Nose–Hoover thermostat).^35^ To further explore the system's response under tension, NPT ensembles were conducted at stresses of 0, 0.05, 0.09, 0.10, 0.11, and 0.12 GPa.

## 3 Results and discussion

### 3.1 Biological toxicity of ATBC, ESO, TCP, DOP

**Fig. 2:**
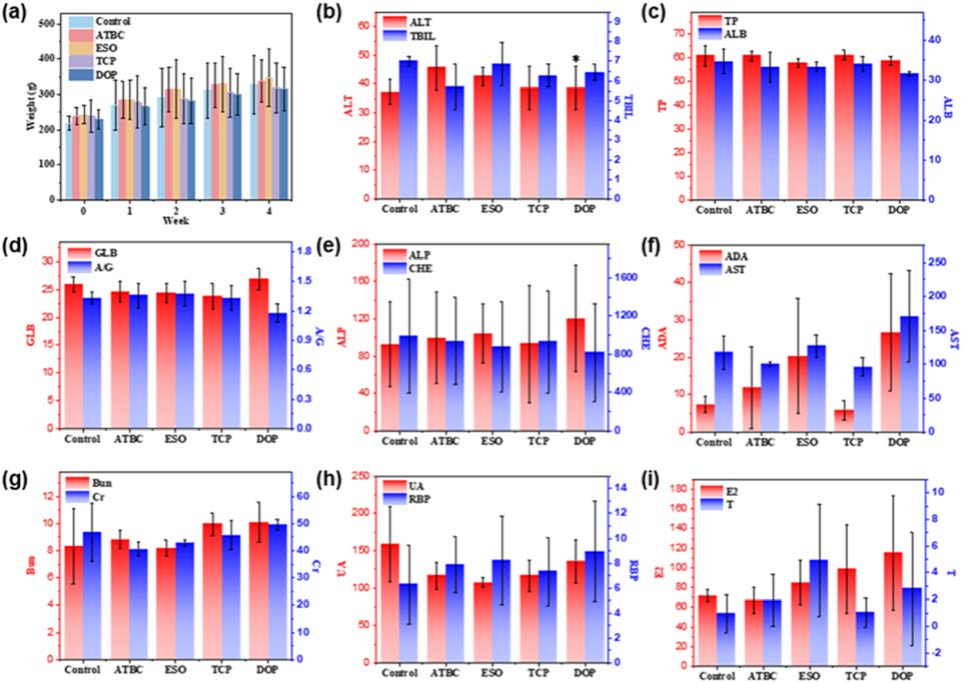
The Effects of four plasticizers (ATBC, ESO, TCP, and DOP) on SD rats in body weight (a), liver function (b–f), renal function (g and h) and sex hormone levels (i) (significant difference, *P < 0.05).

To evaluate the effects of ATBC, ESO, TCP, and DOP, on blood toxicity such as kidney function, liver function, and sex hormones of SD rats, a 28 days toxicological study is conducted by administering them orally to SD rats. The specific results are shown in Fig. 2. After 28 days of gavage, SD rats did not exhibit any abnormal phenomena such as poisoning, injury, or death. Visual observation shows that all rats are in good condition, with normal bowel movements and shiny hair. Analyzing the results (Fig. 2(a)), no statistically significant difference is observed in the body weight index of SD rats given four kinds of plasticizers compared with the control group. All SD rats exhibit weight gain, with ESO plasticizer administered orally showing the fastest weight gain, followed by ATBC administered orally. In contrast, the weight gain of SD rats treated with TCP and DOP plasticizers by gavage is slower and lower than that of the control group.

According to Fig. 2(b–f), the impact of four plasticizers on the liver function of SD rats is as follows: compared to the control group, SD rats administered with these four plasticizers did not exhibit significant differences in indicators such as total bilirubin (TBIL), total protein (TP), albumin (ALB), globulin (GLB), albumin/globulin ratio (A/G), alkaline phosphatase (ALP), cholinesterase (CHE), adenosine deaminase (ADA), and aspartate aminotransferase (AST). However, SD rats administered with DOP plasticizer *via* gastric gavage showed significant differences in the aspartate aminotransferase (ALT) indicator (*P < 0.05). This suggests that DOP plasticizer has an impact on the liver function of rats.^36^ According to the results shown in Fig. 2(g and h), the impact of four plasticizers on the renal function of rats is as follows: compared to the control group, SD rats administered with these four plasticizers *via* gastric gavage did not exhibit significant differences in indicators such as urea (Bun), creatinine (Cr), uric acid (UA), and retinol-binding protein (RBP). This suggests that the four plasticizers do not significantly affect the renal function of SD rats. Estrogen (E2) and testosterone (T) are important hormonal indicators for assessing female and male reproductive capabilities. According to the results shown in Fig. 2(i), the impact of four plasticizers on the hormonal levels of SD rats is as follows: compared to the control group, SD rats administered with these four plasticizers *via* gastric gavage did not exhibit significant differences in indicators such as E2 and T. This suggests that the four plasticizers do not significantly affect the hormonal balance in SD rats. In summary, although gender differences and individual variations may lead to significant errors in blood routine indicators, based on the blood routine indicators of SD rats, we tentatively infer that oral administration of ATBC, ESO, and TCP for 28 days does not result in hematotoxicity. However, after 28 days of oral administration, DOP plasticizer does cause certain damage to the liver function of SD rats.

**Fig. 3:**
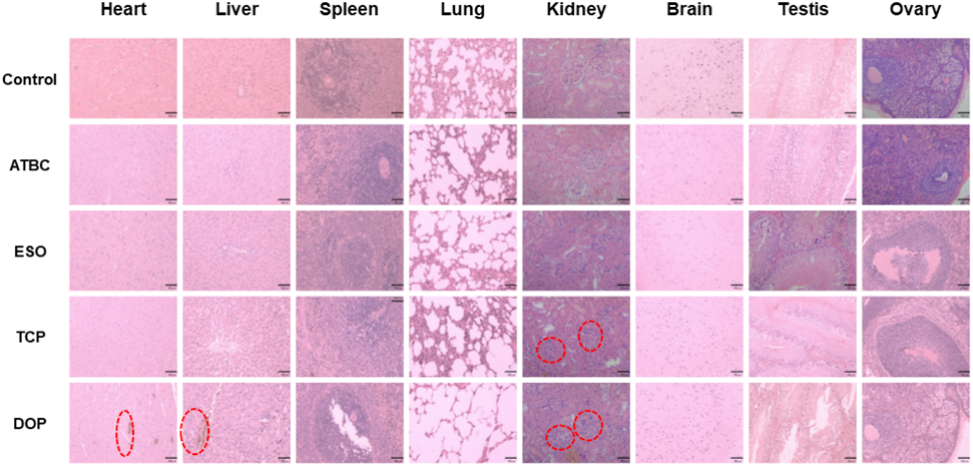
The Effects of four plasticizers (ATBC, ESO, TCP, and DOP) on tissues and organs of SD rats.

To further investigate the safety of ATBC, ESO, TCP, and DOP plasticizers, the tissues and organs of SD rats (as shown in Fig. 3) is analyzed. The study reveals that the DOP group exhibit significant pathological changes, including abnormal cardiac muscle structure, increased myocardial spacing, interstitial congestion, hemorrhage in the lungs, pulmonary expansion, narrowed interstitium, and enlarged alveolar pores, as well as a significant decrease in sperm count in testicular tissue. Additionally, both the DOP and TCP groups exhibit notable hepatocyte proliferation, slightly widened hepatic sinusoids, inflammatory cell infiltration in the portal area, increased lymphocytes, and intracellular inflammatory changes consistent with nodular inflammation. The glomerular structure is indistinct, accompanied by inflammatory cell infiltration. In contrast, the control, ATBC and ESO groups show normal organ tissue. For instance, cardiac muscle fibers display uniform staining, clear striations, and no signs of degeneration or necrosis. The hepatic lobular structure appears normal, with well-defined hepatocyte morphology, orderly arrangement, and no evidence of sinusoidal congestion. The spleen exhibits distinct red and white pulp structures, with normal lymphoid follicles. Lung bronchioles have clear structures, intact alveoli, no significant dilation, and a healthy appearance. The gray and white matter of the brain display clear structures, with normal neuronal morphology and no signs of inflammatory cell infiltration. Furthermore, in all groups, ovarian follicles at various stages exhibit clear morphological structures, without degeneration, necrosis, or inflammatory cell infiltration in the stroma. Similarly, the seminiferous tubules in the testes show well-arranged germ cells at different developmental stages, maintaining normal morphology.

Based on the blood routine indicators and liver tissue pathology analysis in SD rats, we can draw the following conclusions: the biocompatibility ranking of DOP, ATBC, ESO, and TCP is as follows: ESO > ATBC > TCP > DOP. These findings are crucial for assessing the potential toxicity of plasticizers and selecting appropriate alternatives.

### 3.2 Structure and characterization of PVC-based composites

**Fig. 4:**
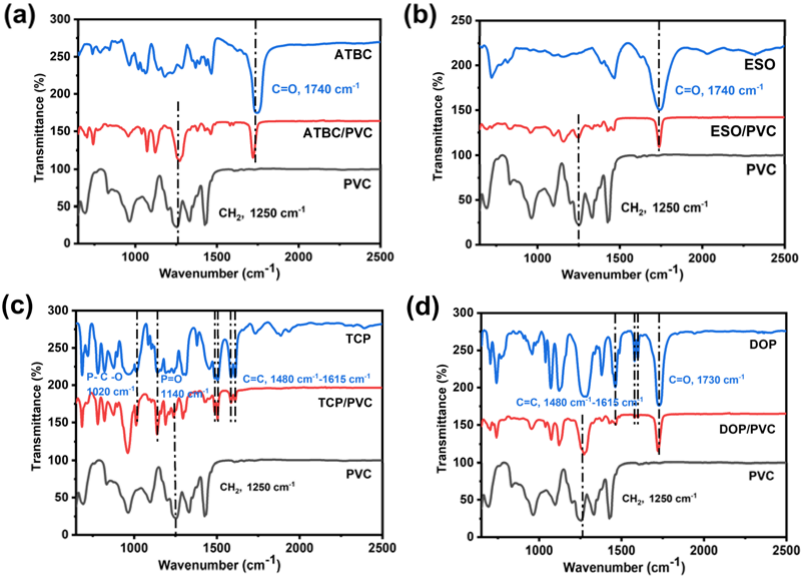
FTIR curves of PVC, plasticizer ((a) ATBC, (b) ESO, (c) TCP and (d) DOP), plasticizer/PVC composites.

The addition of plasticizers to the PVC matrix affects the molecular structure and thermal stability of the resin. The FT-IR spectra of ATBC/PVC, ESO/PVC, TCP/PVC and DOP/PVC (50% wt%) are indicated in Fig. 4(a–d). These spectra exhibit characteristic peaks of both PVC and the respective plasticizers. For instance, a prominent absorption peak is observed around 1250 cm^−1^, representing the CH_2_ group in PVC.^37^ In the cases of ATBC/PVC and ESO/PVC, a new strong peak emerges around 1740 cm^−1^ belonged to the tensile vibration of C=O, which is associated with ATBC and ESO, alongside the CH_2_ peak of PVC. In the case of TCP/PVC, new absorption peaks are evident at 1020, 1140, and 1480 to 1615 cm^−1^, attributed to the vibration absorption peaks of P–O–C, P=O, and the benzene ring characteristic of TCP.^38^ For DOP/PVC, the peaks of C=O and benzene ring are still existed. The above results demonstrate that ATBC, ESO, TCP and DOP are successfully dispersed in PVC resin. In addition, the characteristic peaks of plasticizer/PVC are just a simple superposition of PVC and plasticizers. Therefore, there is no chemical bond formation between PVC and plasticizer, but simply intermolecular forces.

**Fig. 5:**
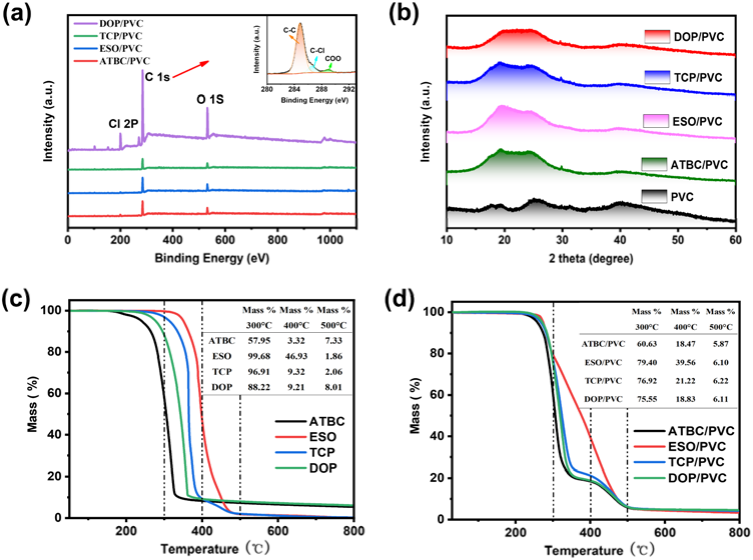
(a) XPS, (b) XRD and (c and d) TG curves of PVC, plasticizer (ATBC, ESO, TCP and DOP), plasticizer/PVC composites.

Similar results were also obtained with XPS.^39^ As shown in Fig. 5(a), when ATBC, ESO, TCP, and DOP plasticizers are added to PVC, the XPS spectra of ATBC/PVC, ESO/PVC, TCP/PVC, and DOP/PVC exhibit an O 1s peak near 531.5 eV.^40^ The C 1s peak is also deconvoluted into C–C, C–Cl, and COO peaks, which further indicates that no new chemical bonds were formed after the plasticizers were introduced into the resin.^41^ However, this non-covalent bonding interactions allow the plasticizer to be easily precipitated from the PVC phase.

The XRD patterns of PVC, ATBC/PVC, ESO/PVC, TCP/PVC, and DOP/PVC are shown in Fig. 5(b). For pure PVC film, a broad shallow double diffraction peak is observed between 16 °C and 28 °C, indicating its amorphous nature. After adding ATBC, ESO, TCP, and DOP plasticizers, the shallow double peaks in the PVC composites become less distinct, suggesting that the plasticizers disrupt the regularity of PVC. Thermal stability serves as a pivotal indicator in assessing the performance of composites. Fig. 4(c and d) shows the TG curves of ATBC, ESO, TCP, DOP and their corresponding PVC composites. It is found that ESO has the best thermal stability followed by TCP, DOP and ATBC, where the superior stability of ESO is attributed to its longer molecular chain structure. Similarly, the stability of the plasticizer/PVC composites is still ESO/PVC > TCP/PVC > DOP/PVC > ATBC/PVC. In general, the mass loss of PVC resin consists of two stages: in the first stage (200–400 °C), the hydrochloric acid leaves rapidly due to the generation of chlorine radicals. In the second stage (400–600 °C), the structure of the PVC resin is rearranged.^42^ It is evident that ESO, acting as a plasticizer, effectively retards the formation of chlorine radicals in PVC resin, consequently enhancing the stability of the resin.

### 3.3 Antisolvent properties and mechanical and of PVC-based composites

**Fig. 6:**
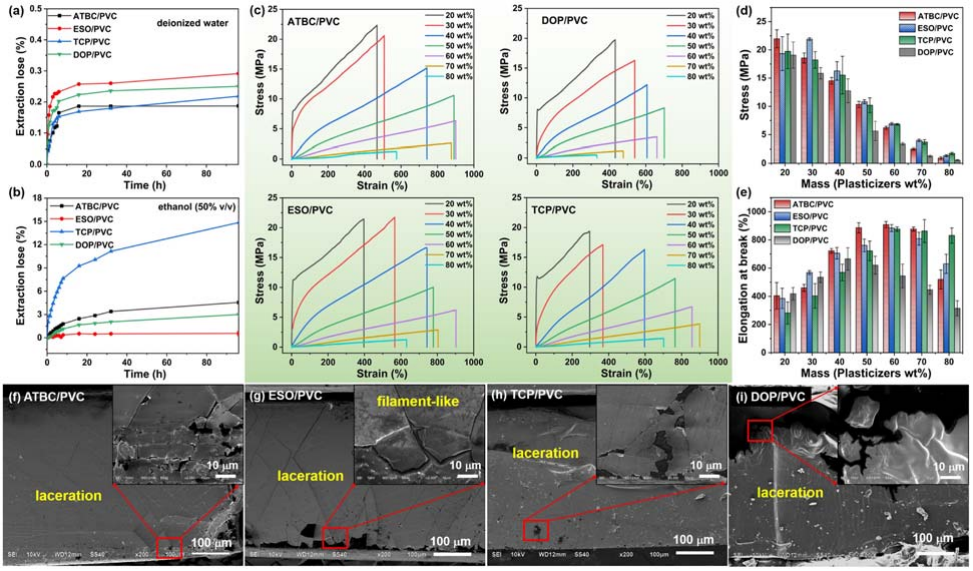
(a and b) Mobility of plasticizers in PVC composite film in two solvents. (c) Typical stress–strains curves of plasticizer/PVC films with different amounts of plasticizers. (d and e) The elongation at break and tensile strength of PVC-based films. (f–i) SEM of the fracture surface of ATBC/PVC, ESO/PVC, TCP/PVC and DOP/PVC (the plasticizer content is 50 wt%).

The stability of plasticizers in PVC is a critical indicator for assessing its performance. As illustrated in Fig. 6(a and b), two solvents are chosen for investigating plasticizer mobility test. The migration rates of plasticizers follow the order MESO/PVC > MDOP/PVC > MTCP/PVC > MATBC/PVC in distilled water, and MTCP/PVC > MATBC/PVC > MDOP/PVC > MESO/PVC in 50% (v/v) ethanol. It is evident that the migration rate of plasticizers in deionized water is significantly lower than that in 50% (v/v) ethanol. Among them, the migration rate of TCP in 50% (v/v) ethanol is very high, with a migration rate of up to 15% after 96 h, which means that 30 wt% of TCP precipitates from the PVC resin phase. This high migration phenomenon can seriously damage the flexibility of the resin, making it harder. While, compared with ESO and ATBC, the migration rate of DOP is not very large, but its toxicity is relatively high. However, ATBC is a plasticizer suitable for the aqueous applications, while ESO is suitable for the oil phase.

The stress–strain curves of ATBC/PVC, ESO/PVC, TCP/PVC, and DOP/PVC are depicted in Fig. 6(c). The tensile strength of the plasticizer/PVC decreases gradually with the increase in plasticizer content, as the plasticizer disrupts the cross-linking between the PVC chains. As expected, the elongation at break of PVC increases with increasing plasticizer content, reaching optimal values at 50–70%, and then decreases. As depicted in Fig. 6(d), while the tensile strength of these four materials decreases with increasing plasticizer content, ESO/PVC demonstrates relatively superior tensile strength compared to the others, whereas DOP/PVC exhibits inferior performance. The elongation at break (Fig. 6(e)) of ATBC/PVC, ESO/PVC, and TCP/PVC is superior to that of DOP/PVC, with maximum elongations at break of 908.6% (ATBC, 60 wt%), 884.1% (ESO, 60 wt%), 876.9% (TCP, 60 wt%), and 665.7% (DOP, 40 wt%), respectively. While, when the content of plasticizer is 40 wt%, the elongation at break is 723.0% (ATBC/PVC), 705.7% (ESO/PVC), 569.7% (TCP/PVC) and 665.7% (DOP/PVC), respectively, and the elongation at break of TCP/PVC is less than that of DOP/PVC. Therefore, considering the dosage of plasticizer, TCP may not be the preferred choice. However, from the perspective of plasticizer performance and dosage, ATBC and ESO are better alternative plasticizers. Among them, ATBC is more suitable for applications that require higher material toughness, and ESO is more suitable for applications that require better material mechanical strength.

SEM of the cross-sectional fracture surfaces of ATBC/PVC, ESO/PVC, TCP/PVC, and DOP/PVC films (with plasticizer content at 50 wt%) is depicted in Fig. 6(f–i). The pure PVC appears relatively flat with minor roughness, suggesting a predominantly brittle behavior with minimal plastic deformation (Fig. S2†). However, upon the addition of plasticizers, all plasticizer/PVC films demonstrate notable signs of plasticization. Their fracture surfaces exhibit corrugated and sponge-like morphologies, often with filamentous structures. This observation indicates that in ATBC/PVC, ESO/PVC, TCP/PVC, and DOP/PVC, the plasticizers exhibit pronounced plasticizing effects with no apparent phase separation, signifying excellent compatibility between the plasticizers and PVC.

**Fig. 7:**
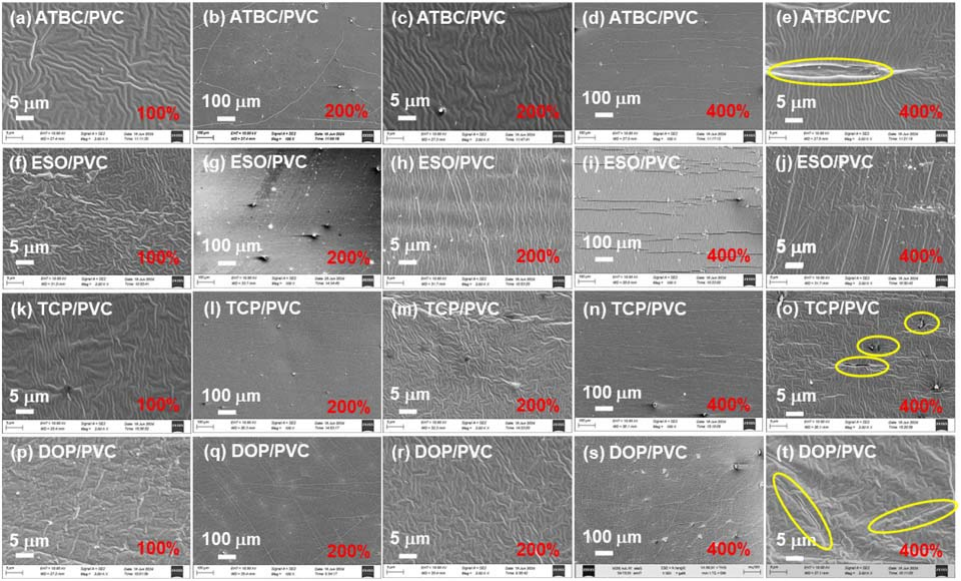
(a) ATBC/PVC (elongation 100%), (b and c) ATBC/PVC (elongation 200%), (d and e) ATBC/PVC (elongation 400%), (f) ESO/PVC (elongation 100%), (g and h) ESO/PVC (elongation 200%), (i and j) ESO/PVC (elongation 400%), (k) TCP/PVC (elongation 100%), (l and m) TCP/PVC (elongation 200%), (n and o) TCP/PVC (elongation 400%), (p) DOP/PVC (elongation 100%), (q and r) DOP/PVC (elongation 200%) and (s and t) DOP/PVC (elongation 400%) SEM image of composites (plasticizer content 50% wt%) at static stretching for 15 days.

In order to evaluate the long-term mechanical fatigue performance of different plasticizer/PVC composites, SEM observations are conducted on PVC-based composites (*M*_PVC_ = 50 wt%) with elongation of 200% and 400% after static holding for 15 days, as shown in Fig. 7. The unstretched plasticizer/PVC composites (with an elongation of 100%) exhibits an obvious wrinkled morphology in the microstructure. As the degree of stretching increases, the number of cracks in plasticizer/PVC composite materials significantly rises, along with increased crack propagation, while wrinkles decrease. The observed wrinkling and necking phenomena confirm that the deformation of all PVC-based composites is due to plastic deformation. Specifically, the ESO/PVC (Fig. 7(f–j)) exhibits the most surface cracks, indicating the most severe degree of damage. This is followed by the DOP/PVC (Fig. 7(p–t)). The cracks in ATBC/PVC (Fig. 7(a–e)) and TCP/PVC (Fig. 7(k–o)) composites are relatively few, although ATBC/PVC also displays an obvious necking phenomenon. Additionally, pores are present on the surface of TCP/PVC, in addition to the necking phenomenon. Overall, except for ESO/PVC, this trend aligns with the short-term mechanical performance observed in PVC-based composites (Fig. 6). Consequently, it can be inferred that the long-term endurance fatigue performance of ESO/PVC composite materials is poor.

In order to further evaluate the long-term compatibility of the plasticizer with PVC resin, SEM and energy dispersive diffraction (EDS) of plasticizer/PVC composites (*M*_PVC_ = 50 wt%) with a tensile strength of 400% are studied under static conditions for 15 days, as shown in Fig. S3.† ATBC/PVC (Fig. S3(a–d)†), ESO/PVC (Fig. S3(e–h)†), and DOP/PVC (Fig. S3(m–p)†) exhibit uniform surface distribution, while TCP/PVC (Fig. S3(i–l)†) also displays uniform distribution. These results indicate that the composition of PVC matrix composites remains uniform, and no phase separation occurs even after long-term static stretching. Consequently, it can be inferred that ATBC, ESO, TCP and DOP all have excellent compatibility with PVC resin and are not affected by tensile damage.

### 3.4 Compatibility of ATBC, ESO, TCP and DOP with PVC resin

**Fig. 8:**
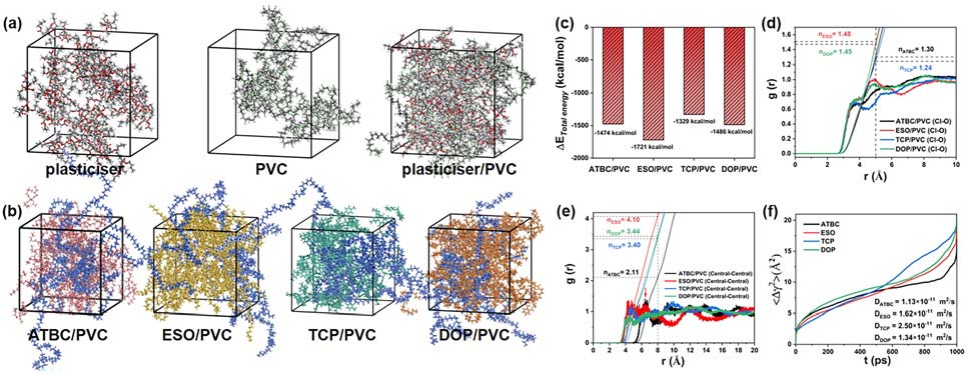
(a) Microstructure of plasticizer, PVC, plasticizer/PVC. (b) Microstructure of ATBC/PVC, ESO/PVC, TCP/PVC, DOP/PVC. (c) Δ*E* of ATBC/PVC, ESO/PVC, TCP/PVC, DOP/PVC. (d) RDFs and coordination number of Cl atoms of PVC to O atoms of plasticizers. (e) RDFs and coordination number between central atoms of ATBC, ESO, TCP and DOP. (f) MSDs of ATBC, ESO, TCP and DOP in PVC.

The microstructure of plasticizer, PVC and plasticizer/PVC shows that PVC has good compatibility with ATBC, ESO, DOP and TCP (Fig. 8(a and b)). To better explain the compatibility between different plasticizers and PVC, MD simulations are used to statistically analyze the total energy change (Δ*E*) during the formation of composites between plasticizers and PVC (PVC + plasticizer → plasticizer/PVC). Here, Δ*E* is defined as Δ*E* = *E*_plasticizer/PVC_ − *E*_plasticizer_ − *E*_PVC_.^43^ As depicted in Fig. 8(c), the Δ*E* values for ATBC/PVC, ESO/PVC, TCP/PVC, and DOP/PVC are −1474, −1721, −1329, and −1486 kcal mol^−1^, respectively. Based on thermodynamic principles, if Δ*E* < 0, the dispersion behavior of plasticizers can occur spontaneously. Therefore, ATBC, ESO, TCP, and DOP can all spontaneously disperse in PVC resin. Among them, Δ*E* for ESO/PVC is the most negative, indicating that ESO exhibits the best compatibility with PVC, followed by DOP, ATBC, and TCP.

To further investigate the interaction between PVC and plasticizers, the radical distribution functions (RDFs) of Cl atoms of PVC to O atoms of plasticizers are conducted and shown in Fig. 8(d). Compared with ATBC, DOP and TCP, the distribution of Cl atoms around O atoms of ESO is the highest. The number of O atoms within the range of 0–5 Å from the Cl atoms were further calculated, and the coordination numbers were calculated to be 1.30 (ATBC), 1.48 (ESO), 1.24 (TCP) and 1.45 (DOP), respectively. Therefore, the interaction between PVC and ESO is the strongest, followed by DOP, ATBC and TCP. This strong interaction is also the main reason for the good compatibility of ESO in PVC.

The plasticizing efficiency of plasticizers is not only related to their resin compatibility, but also influenced by their self-aggregation effect. In other words, if the plasticizers tend to aggregate severely in the resin, these agglomerated sites will become fracture points during the stretching process, leading to a decrease in the mechanics performance of the PVC. To elucidate the aggregation behaviour of plasticizers in resin, an atom in the plasticizers was labeled as a central atom (Fig. S4†) and the RDFs and coordination numbers between these central atoms were calculated. As shown in Fig. 8(e), from 0 Å to 8.0 Å, the coordination numbers of plasticizers are 2.11 (ATBC), 4.10 (ESO), 3.40 (TCP) and 3.44 (DOP), respectively. It is obvious that ESO is the most prone to self-aggregation in PVC resin, followed by DOP, TCP and ATBC. Among them, the aggregation of ESO is related to their longer alkyl chains, while the difficulty of aggregation of ATBC is attributed to their tetrahedral structure (steric hindrance). In addition, although the molecules of DOP and TCP are not large, there are no obvious aggregation peaks in their RDFs. This may be the significance of introducing benzene rings into the molecular structure of plasticizers, which can effectively prevent self-aggregation.

Solvent resistance is a crucial factor in evaluating the performance of plasticizers, influenced by various factors including the interaction between plasticizers and PVC, agglomeration of plasticizers, volume of plasticizers, and the choice of solvent. Fig. 8(f) shows the mean square displacements (MSDs) of plasticizers in PVC resin, the diffusion coefficients of plasticizers are 1.13 × 10^−11^ (ATBC), 1.62 × 10^−11^ (ESO), 2.50 × 10^−11^ (TCP) and 1.34 × 10^−11^ m^2^ s^−1^ (DOP), respectively.^44^ Regardless of the selected solvent environment, the stability of these four plasticizers in PVC resin is TCP ≪ ESO < DOP < ATBC.

### 3.5 Microscopic plasticization mechanism of ATBC, ESO, TCP and DOP

**Fig. 9:**
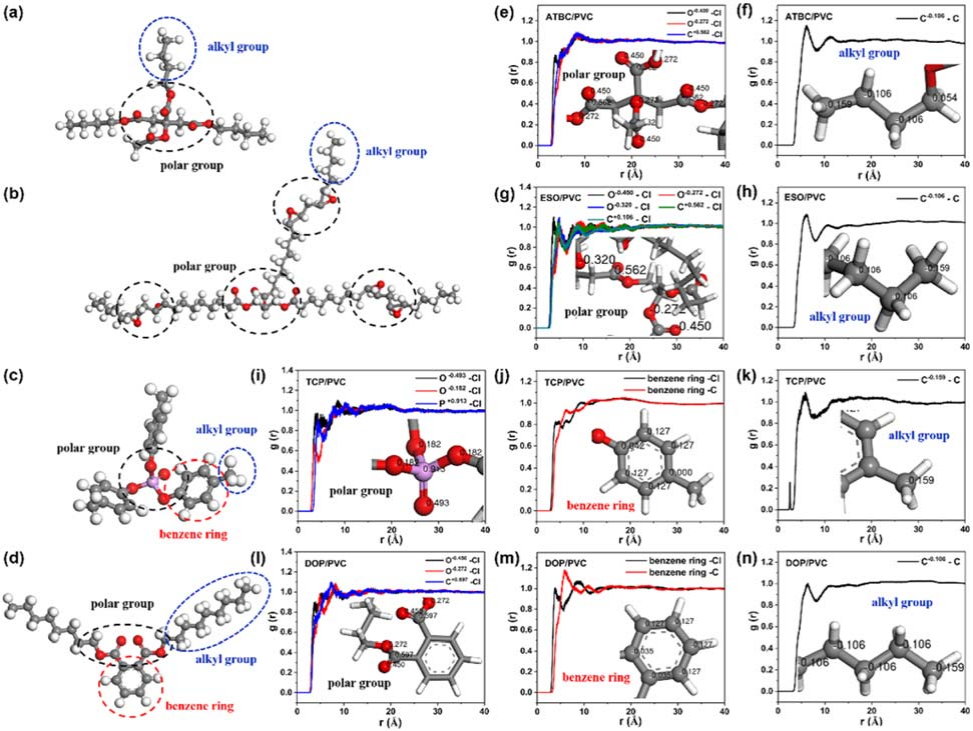
(a–d) Schematic diagrams of ATBC, ESO, TCP and DOP for splitting. (e) RDFs of Cl (PVC) around the polar group of ATBC. (f) RDFs of C (PVC) around the alkyl group of ATBC. (g) RDFs of Cl (PVC) around the polar group of ESO. (h) RDFs of C (PVC) around the alkyl group of ESO. (i) RDFs of Cl (PVC) around the polar group of TCP. (j) RDFs of C (PVC) around the benzene ring of TCP. (k) RDFs of C (PVC) around the alkyl group of TCP. (l) RDFs of Cl (PVC) around the polar group of DOP. (m) RDFs of C (PVC) around the benzene ring of DOP. (n) RDFs of C (PVC) around the alkyl group of DOP.

To explore the mechanism of action between PVC and plasticizers, the plasticizers are disassembled in Fig. 9(a–d). For ATBC, it is divided into two parts: black circle-polar groups (O and C atoms on the ester group, O^−0.45^, O^−0.272^ and C^+0.562^), whose main interaction with PVC are van der Waals and electrostatic forces, and blue circle-hydrophobic alkyl groups (C^−0.106^), which interact with PVC in a hydrophobic association. Further statistics are conducted on the RDFs between the above two types of groups and Cl and C on the PVC. As depicted in Fig. 9(e) and (f), the distribution of Cl around the polar groups is not very high, while there is a distinct C distribution peak around the alkyl chain. Due to the large spatial site resistance of ATBC, it is relatively difficult to form stable dipole pairs between ATBC and PVC by van der Waals force or electrostatic interaction, and the interaction of ATBC with PVC may depend more on the intertwining of hydrophobic alkyl chains with PVC chains. Therefore, appropriately increasing the chain length of the hydrophobic alkyl chains of citrate plasticizers is beneficial to increase their compatibility with PVC.

Similar to ATBC, ESO can be simply broken down into two parts: black circle-polar groups (O and C atoms on the ester group and epoxy group, O^−0.45^, O^−0.272^, O^−0.320^, C^+0.562^ and C^+0.106^) and blue circle-alkyl groups (C^−0.106^). As shown in Fig. 8(g and h), Cl has a higher distribution peak around the polar groups and the ESO alkyl chains also has a larger distribution peak around the PVC. Thus, the interaction between ESO and PVC includes van der Waals forces and electrostatic interactions among polar groups, as well as mutual entanglement between alkyl chains due to hydrophobic interactions, which explains the better bonding and compatibility of ESO with PVC.

As shown in Fig. 9(i–n), both TCP and DOP contain benzene rings, which in turn divided these two plasticizers into three parts, the polar groups (black circle, O^−0.493^, O^−0.182^ and P^+0.913^ of TCP, and O^−0.450^, O^−0.272^ and C^+0.597^ of DOP), the benzene rings (red circle) and the alkyl groups (blue circle, C^−0.159^ and C^−0.106^). It is not difficult to find that the interaction force between the polar groups in these two plasticizers is not strong and the distribution of PVC around the benzene rings is also not high, indicating that the polar groups and benzene rings do not contribute much to the improvement of TCP and DOP compatibility. The alkyl chains of both TCP and DOP have a larger distribution peak around PVC, indicating that hydrophobicity is still the main binding force between these two plasticizers and PVC. However, due to having only one alkyl chain, TCP exhibits the poorest compatibility and solvent resistance in PVC resin compared to other plasticizers, making it more prone to precipitation from the bulk phase.

### 3.6 Tensile failure mechanism of plasticizer/PVC composites

**Fig. 10:**
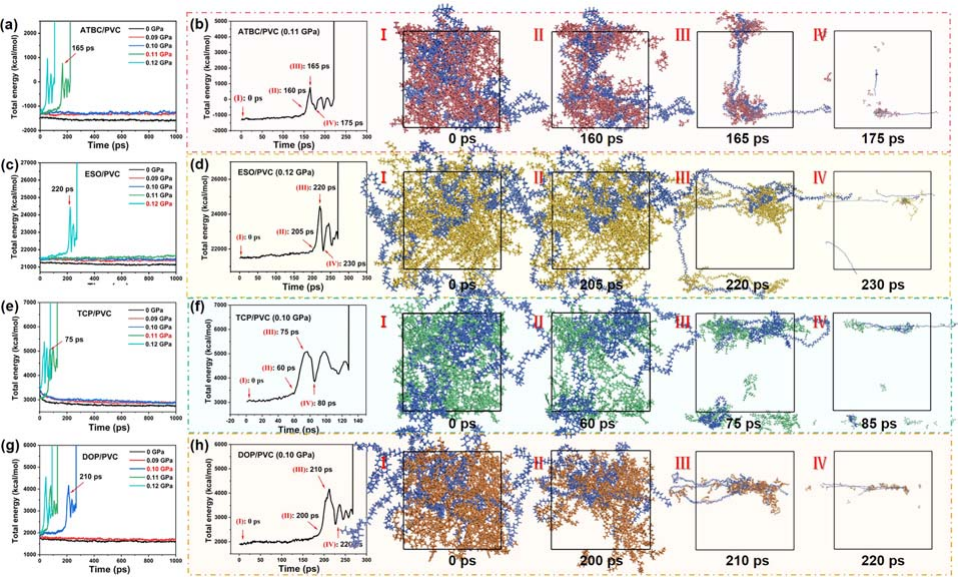
Total energy of (a and b) ATBC/PVC, (c and d) ESO/PVC, (e and f) TCP/PVC and (g and h) DOP/PVC under varying stress levels.

To delve into the tensile failure mechanism of PVC-based composite films, as illustrated in Fig. 10, the total energy of ATBC/PVC, ESO/PVC, TCP/PVC, and DOP/PVC under stress levels ranging from 0 to 0.12 GPa was examined. The total energy was determined using the following formula:*E*_total energy_ = *E*_kinetic energy_ + *E*_potential energy_where the *E*_potential energy_ is contained *E*_bonds_, *E*_angles_, *E*_dihedrals_, *E*_cross_ and *E*_non-bond_. It is not difficult to find that the PVC composites all show a sudden increase in *E*_total energy_ as the tensile strength increases. The inflection points of total energy correspond to the changes of state of the composites in the simulated system, which can be simply distinguished between elastic deformation and plastic deformation regions in different tensile environments. In the case of ATBC/PVC (Fig. 10(a)), for instance, as the stress increases from 0 GPa to 0.10 GPa, the total energy of the system undergoes minimal change, despite fluctuations in tensile strength. Consequently, during this phase, the composite remains in the elastic deformation stage. However, with further escalation of tensile strength, there is a sudden surge in the total energy of the system, resulting in severe damage to the composite structure, indicative of plastic deformation. Specifically, the maximum stress of ATBC/PVC (Fig. 10(a and b)) under elastic deformation corresponds to its tensile strength of 0.10 GPa. Similarly, the tensile strength of ESO/PVC (Fig. 10(c and d)), TCP/PVC (Fig. 10(e and f)) and DOP/PVC (Fig. 10(g and h)) can be counted as 0.11, 0.10 and 0.09 GPa, respectively. The time points at which the first plastic deformation of the composites (as indicated by the red arrows) occurred are 0.11 GPa, 165 ps (ATBC/PVC), 0.12 GPa, 220 ps (ESO/PVC), 0.11 GPa, 75 ps (TCP/PVC) and 0.10 GPa, 210 ps (DOP/PVC), respectively. Therefore, according to the theoretical results, the tensile strength of the plasticizer/PVC composites should be ESO/PVC > ATBC/PVC > TCP/PVC > DOP/PVC, which is basically consistent with the data of tensile strength as mentioned before.

## 4 Conclusions

In this study, the biotoxicity of ATBC, ESO, TCP, and DOP was assessed *via* gavage in SD rats. The plasticizing properties and stability of ATBC, ESO, TCP and DOP in PVC resin were investigated using MD simulations and experiments. Biocompatibility ranking of plasticizers in SD rats based on weight, blood routine indicators, and tissue and organ pathology analysis: ESO demonstrated the highest biocompatibility, followed by ATBC, TCP, and DOP. These findings are crucial for evaluating the potential toxicity of plasticizers and selecting appropriate alternatives. Taking into account the mechanical properties of PVC films, the amount of and migration rates of plasticizers, ATBC and ESO are better alternative plasticizers for DOP. Among them, ATBC is more suitable for systems requiring high toughness and aqueous applications, while ESO is more suitable for systems requiring high mechanical strength and oil-based applications. However, ESO exhibits inadequate long-term mechanical fatigue resistance. Meanwhile, MD results indicate that the compatibility of plasticizers with PVC is ESO > DOP > ATBC > TCP, which is related to the interaction force between plasticizers and PVC. For ATBC/PVC, DOP/PVC and TCP/PVC, the interactions are mainly due to the entanglement of their hydrophobic alkyl chains with the PVC through hydrophobic interaction. For ESO/PVC, the interactions involve both van der Waals forces and electrostatic forces between the polar groups as well as intertwining of the alkyl chains.

In summary, ATBC emerges as the optimal environmentally friendly alternative to DOP. As the soft PVC application market expands, enhancing the interaction force and compatibility between citrate plasticizers and PVC could involve judiciously increasing the chain length of the hydrophobic alkyl group in citrate plasticizers.

## Data availability

Data are available on request from the authors.

## Conflicts of interest

There are no conflicts to declare.

## Supplementary Material

RA-015-D4RA07258H-s001
