# Supplementary material for: Comparative analysis of bio-based plasticizers: biocompatibility, plasticizing mechanisms, and molecular dynamics insights
Source: RSC Adv. 2025 Feb 10;15(6):4487–95. doi: 10.1039/d4ra07258h (PMC11808658; doi:10.1039/d4ra07258h)
Supplement: RA-015-D4RA07258H-s001 [file RA-015-D4RA07258H-s001.pdf]

Supplementary Information (SI) for RSC Advances.
This journal is © The Royal Society of Chemistry 2025

Comparative Analysis of Bio-based Plasticizers: Biocompatibility, Plasticizing Mechanisms, and Molecular Dynamics Insights

Li Gao [a, b], Rui Yuan [*b], Lijuan Qiao [c], Chang Tu [b], Rui Tan [b], Shiai Xu [*a, b]

[a] *Shanghai Key Laboratory of Advanced Polymeric Materials, Key Laboratory for Ultrafine Materials of Ministry of Education, School of Materials Science and Engineering, East China University of Science and Technology, Shanghai 200237, China*

[b] *Salt Lake Chemical Engineering Research Complex, Qinghai University, Xining 810016, China*

[c] *Research Center of Basic Medical Science, Medical College, Qinghai University, Xining 810016, China*

Supporting Information available See DOI: 10.1039/x0xx00000x

2.1 Materials

PVC was provided by Shandong Langhui Petrochemical Co., Ltd (Shandong, China). Four different plasticizers were procured from Shanghai Aladdin Biochemical Technology Co., Ltd (Shanghai, China). Tetrahydrofuran (THF) was sourced from Sinopharm Chemical Reagent Co., Ltd (Shanghai, China). Carboxymethylcellulose Sodium (CMC-Na) was purchased from Tianjin Kemeiou Chemical Reagent Co., Ltd. Pentobarbital Sodium was acquired from Beijing Fangcheng Biological Co., Ltd. Formalin Solution was obtained from Servicebio in Wuhan (Product Code: G1101). Hematoxylin and Eosin (HE) Staining was purchased from Solarbio in the United States (Product Code: G1120). Other materials were utilized as received without the need for additional purification.

2.2 Characterizations

The morphologies of plasticizer/PVC surface and their corresponding fracture surface were conducted by 3D profilometer (Super View W1, China) and SEM (JSM-6610LV, Japan). Fourier-transform infrared spectra (FTIR) were obtained using an FTIR spectrometer (Nicolet 6700, USA) within the range of 400-4000 cm^{-1}, with a resolution of 0.5 cm^{-1}. Thermogravimetric analysis (TGA) was conducted utilizing a TGA instrument (STA 449 F3, Germany).

Table S1 Details of ATBC/PVC, ESO/PVC, TCP/PVC and DOP/PVC simulation systems.

Designation	Number of molecules (PVC)	Number of molecules (plasticizer)	Cell size (Å^3)	Density (g/cm^3)
$S_{ATBC/PVC}$	4	61	41.85×41.85×41.85	1.123
$S_{ESO/PVC}$	4	32	45.36×45.36×45.36	1.121
$S_{TCP/PVC}$	4	67	43.53×43.53×43.53	1.180
$S_{DOP/PVC}$	4	63	43.76×43.76×43.76	1.138

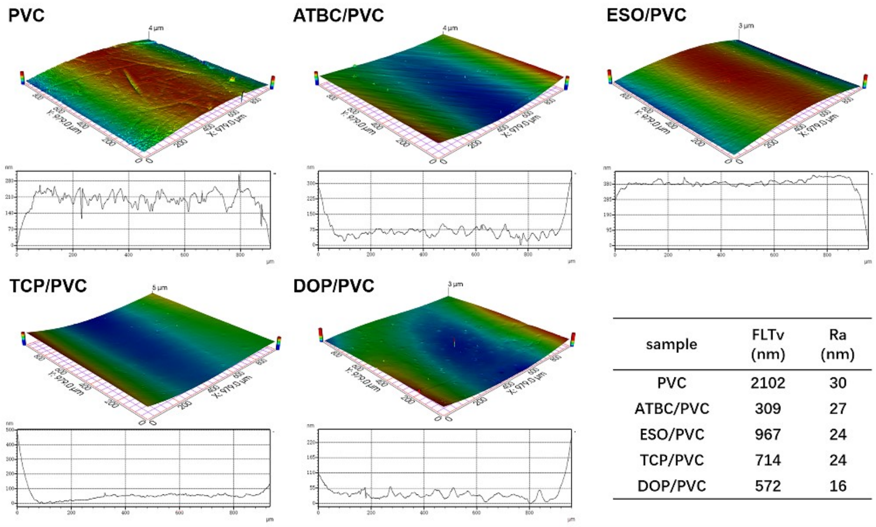

sample	FLTv (nm)	Ra (nm)
PVC	2102	30
ATBC/PVC	309	27
ESO/PVC	967	24
TCP/PVC	714	24
DOP/PVC	572	16

Fig. S1 Surface morphology, flatness and roughness of PVC, ATBC/PVC, ESO/PVC, TCP/PVC and DOP/PVC films.

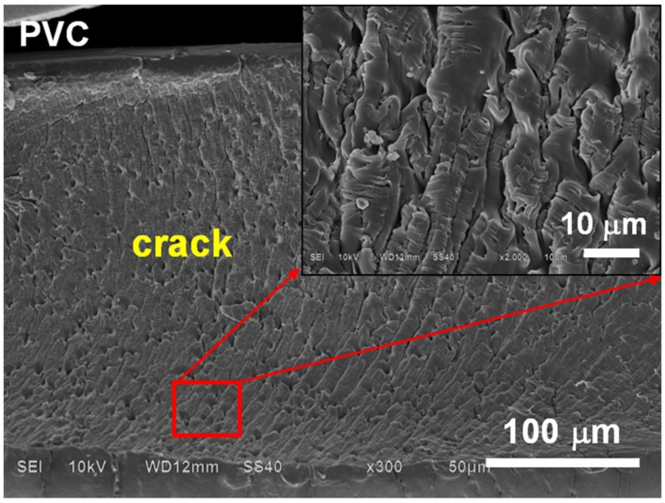

Fig. S2 SEM images of the fracture surface morphologies of PVC.

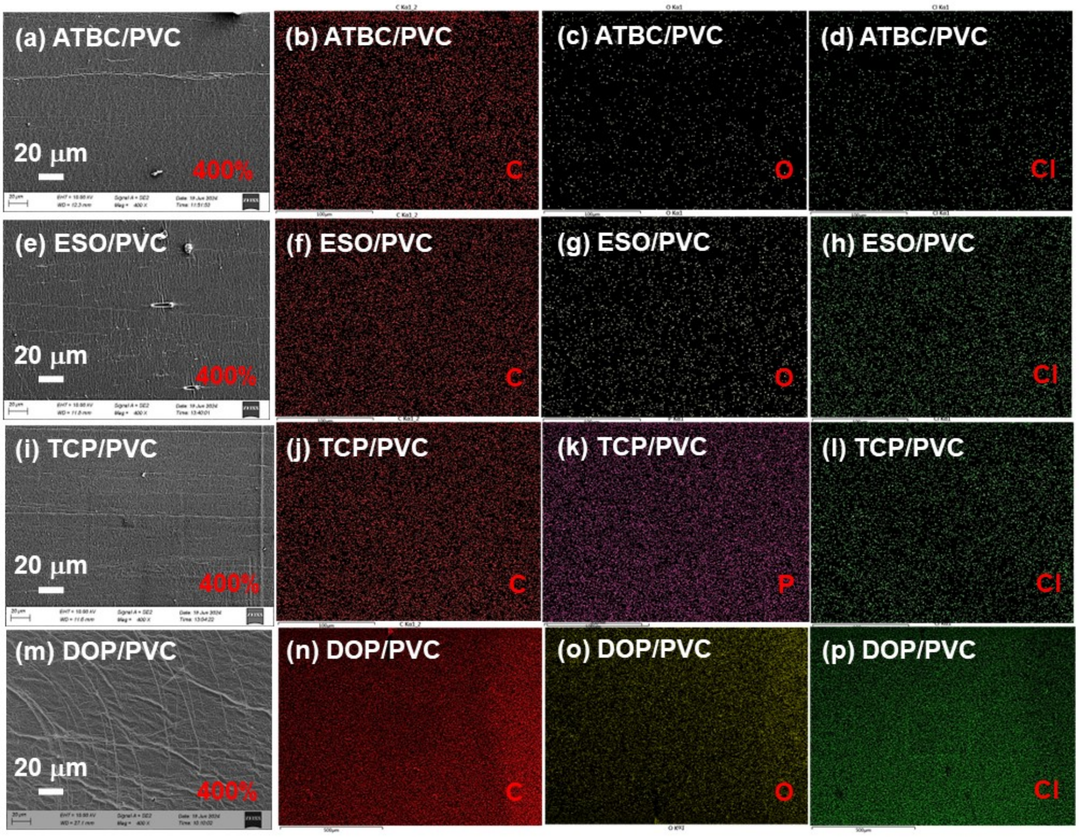

Fig. S3 (a) ATBC/PVC (elongation 400%), (b) ATBC/PVC (C), (c) ATBC/PVC (O), (d) ATBC/PVC (Cl), (e) ESO/PVC (elongation 400%), (f) ESO/PVC (C), (g) ESO/PVC (O), (h) ESO/PVC (Cl), (i) TCP/PVC (elongation 400%), (j) TCP/PVC (C), (k) TCP/PVC (P), (l) TCP/PVC (Cl), (m) DOP/PVC (elongation 400%), (n) DOP/PVC (C), (o) DOP/PVC (O) and (p) DOP/PVC (Cl) SEM and EDS of composites (plasticizer content 50% wt%) at static stretching for 15 d

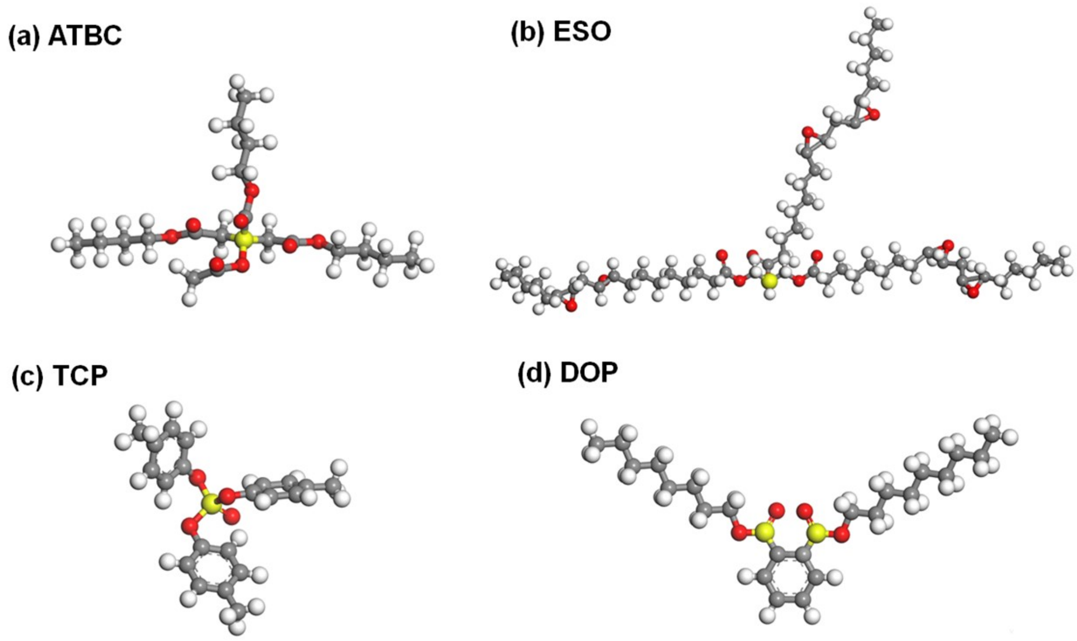

Fig. S4 The central atoms (yellow) of ATBC, ESO, TCP, and DOP.
